# Death Receptor-Induced Apoptosis Signalling Regulation by Ezrin Is Cell Type Dependent and Occurs in a DISC-Independent Manner in Colon Cancer Cells

**DOI:** 10.1371/journal.pone.0126526

**Published:** 2015-05-26

**Authors:** Elisabetta Iessi, Luciana Zischler, Aurélie Etringer, Marion Bergeret, Aymeric Morlé, Guillaume Jacquemin, Alexandre Morizot, Sarah Shirley, Najoua Lalaoui, Selene L. Elifio-Esposito, Stefano Fais, Carmen Garrido, Eric Solary, Olivier Micheau

**Affiliations:** 1 INSERM, U866, Dijon, F-21079, France; 2 UFR des Sciences de Santé, Univ. Bourgogne, Dijon, F-21079, France; 3 Pós-graduação em Ciências da Saúde, Escola de Medicina, Pontifícia Universidade Católica do Paraná, 80215–901, Curitiba, Paraná, Brazil; 4 Department of Therapeutic Research and Medicines Evaluation, Antitumor Drugs Section, Istituto Superiore di Sanita, Viale Regina Elena, Rome, Italy; 5 INSERM, U1009, Villejuif, F-94805, France; 6 Institut Gustave Roussy, Univ. Paris XI, Villejuif, F-94805, France; 7 Centre Georges-François Leclerc, Dijon, F-21000, France; Emory University, UNITED STATES

## Abstract

Ezrin belongs to the ERM (ezrin-radixin-moesin) protein family and has been demonstrated to regulate early steps of Fas receptor signalling in lymphoid cells, but its contribution to TRAIL-induced cell death regulation in adherent cancer cells remains unknown. In this study we report that regulation of FasL and TRAIL-induced cell death by ezrin is cell type dependant. Ezrin is a positive regulator of apoptosis in T-lymphoma cell line Jurkat, but a negative regulator in colon cancer cells. Using ezrin phosphorylation or actin-binding mutants, we provide evidence that negative regulation of death receptor-induced apoptosis by ezrin occurs in a cytoskeleton- and DISC-independent manner, in colon cancer cells. Remarkably, inhibition of apoptosis induced by these ligands was found to be tightly associated with regulation of ezrin phosphorylation on serine 66, the tumor suppressor gene WWOX and activation of PKA. Deficiency in WWOX expression in the liver cancer SK-HEP1 or the pancreatic Mia PaCa-2 cell lines as well as WWOX silencing or modulation of PKA activation by pharmacological regulators, in the colon cancer cell line SW480, abrogated regulation of TRAIL signalling by ezrin. Altogether our results show that death receptor pro-apoptotic signalling regulation by ezrin can occur downstream of the DISC in colon cancer cells.

## Introduction

TNF-Related Apoptosis-Inducing Ligand (TRAIL or Apo2L) induces cell death in a wide variety of cancer cells, but not in normal cells. This peculiarity renders TRAIL and TRAIL derivatives innovative and promising therapeutic agents against malignant diseases. TRAIL triggers cell death upon binding to two transmembrane agonistic receptors: TRAIL-R1 (DR4) [1–3] and TRAIL-R2 (DR5) [1, 2, 4, 5], containing within their intracellular region a Death Domain (DD), which is essential for triggering apoptosis. Activation of TRAIL-R1/TRAIL-R2 allows recruitment of the adaptor protein FADD and proforms of caspase-8 and -10 to form the macromolecular complex called DISC (Death-Inducing Signalling Complex) [6]. Within this complex, caspase-8 and -10 are activated by auto-proteolytic cleavage and released in the cytosol allowing activation of effector caspases [7].

Like TRAIL receptors, Fas, also coined CD95 or APO-1, signals apoptosis through the formation of a DISC [8]. Experimental evidence indicates that Fas linkage to the actin cytoskeleton through ezrin primes human CD4+ T lymphocytes for Fas-mediated apoptosis [9, 10]. CD4 T cell activation, through either HIV-1 gp120 or IL-7, renders CD4 T cells prone to Fas-mediated apoptosis through ezrin-Fas linkage and therefore to apoptosis of bystander uninfected T cells in AIDS patients [11, 12]. In T lymphomas such as Jurkat cells, ezrin was shown to bind Fas, and to be required for cell death triggering [13]. However, ezrin was also suggested, in another study, to inhibit TRAIL- and Fas ligand-induced cell death in T cell lymphomas [14].

Ezrin is a member of the ezrin, radixin, moesin (ERM) family of proteins, that link various integral membrane proteins to the actin cytoskeleton [15]. ERM proteins are usually present in the cytoplasm in an inactive/closed form, in which the amino-terminal membrane protein-binding domain (FERM or N-ERMAD domain) is masked due to its association with the carboxyl, actin-binding domain (C-ERMAD). ERM activation is proposed to occur through phosphorylation and binding of phosphatidylinositol 4,5-bisphosphate (PIP_2_) [16].

Phosphorylation of ezrin on threonine 567 induces a transition to the open/active form, which correlates with its recruitment to the plasma membrane, where it binds membrane molecules. Other phosphorylation sites on ezrin have been described. Phosphorylation on tyrosine residues 145 and 353, e.g. in response to epidermal growth factor, promotes survival [17] and epithelial differentiation [18]. Src-mediated ezrin phosphorylation on tyrosine 145 increases adhesion of epithelial cells to extracellular matrix [19], while phosphorylation of serine 66 by protein kinase A (PKA) is associated with acid secretion in gastric cells [20].

We here further explore the function of ezrin in the TRAIL pathway. We demonstrate that ezrin phosphorylation at serine 66 selectively contributes to TRAIL-induced cell death regulation downstream of the TRAIL DISC in colon cancer cells.

## Materials and Methods

### Ligand production and antibodies

Flag-tagged recombinant soluble human TRAIL, His-tagged TRAIL and Fas ligand were produced and used as described previously [21]. Anti-Flag (M2), 8-bromo-cyclic AMP, Forskolin and orthovanadate were purchased from Sigma-Aldrich (Lyon, France). PKA inhibitor, H89 was from Cayman (Interchim, Montluçon, France). For western blot analysis, anti-TRAIL-R1 and anti-TRAIL-R2 antibodies were purchased from Chemicon (Millipore, Molsheim, France), anti-FADD, anti-phospho-ezrin (Thr567) and anti-moesin were obtained from Transduction Laboratories (BD biosciences, Le Pont de Claix, France), anti-caspase-8 and anti-caspase-10 were from Medical & Biological Laboratories (Clinisciences, Montrouge, France). Antibodies against phospho-ezrin (Thr567)/radixin (Thr564)/moesin (Thr 558), phospho-PKA Substrate (RRXS*/T*) (100G7E), Phospho-(Ser) PKC Substrate (P-S3-101), Phospho-CREB (Ser133) (87G3) and the active cleaved fragment of caspase-3 and caspase-9 were from Cell Signaling (Ozyme). Anti-radixin, caspase-2, GAPDH and HSC-70 were from Santa Cruz Biotechnology (Tebu-bio, Le Perray en Yvelines, France). Anti-actin, anti-ezrin and anti-VSV glycoprotein antibodies were purchased from Sigma-Aldrich (Lyon, France). For flow cytometry experiments, anti-Bax was obtained from BD biosciences. The secondary antibody was an Alexa-488-coupled goat anti-mouse from Molecular Probes (Invitrogen, Cergy Pontoise, France). For immunoprecipitation, the anti-ezrin (clone 3C12), anti-Flag (M2) and anti-VSV glycoprotein (P5D4) antibodies were purchased from Sigma-Aldrich Anti-TRAIL-R1 (wB-S26) and anti-TRAIL-R2 (B-D37) antibodies were provided by Gen-Probe (Diaclone, Besançon, France).

### Cell culture

The HCT116 (human colon carcinoma), SW480 (human colon adenocarcinoma), SK-HEP-1 (human hepatocellular carcinoma), and Mia PaCa-2 cell lines were cultured with high-glucose Dulbecco’s modified Eagle’s medium (Lonza, Levallois-Perret, France) supplemented with 10% fetal bovine serum (Lonza) and penicillin/streptomycin (100 μg/ml of each). PANC-1 (human pancreatic carcinoma) cells were cultured in RPMI 1640 as above. All cell lines were grown in 5% CO_2_ at 37°C.

### Plasmid construction

VSV-tagged ezrin WT was subcloned from pEGFP-N1 vector (Invitrogen) to pCR-3 (Invitrogen). Mutations S66A, S66D, Y145F, Y145D, Y353F, Y353D, T567A, T567D, R579A were created by standard PCR methods and a site-directed mutagenesis kit (Stratagene, La Jolla, CA) according to the manufacturer’s manual (see S1 Table for primer description). The S66D, Y145D, Y353D, T567D were created to mimic phosphorylated ezrin, whereas S66A, Y145F, Y353F, T567A were generated as nonphosphorylatable ezrin. The VSV-tagged ezrin mutants were subcloned into the pMSCV-puro expression vector as HindIII/XhoI fragments. All constructs were confirmed by sequencing.

### Retrovirus production and cell transduction

The retroviral vector pMSCV-puro expression and the generation of viruses have been previously described [22]. HCT116, SW480, MIA PaCa-2 and SK-HEP-1 cells were infected for 16 hours with viral supernatants containing 8 μg/ml polybrene (Hexadimethrin Bromide from Sigma Aldrich), washed in phosphate-buffered saline from Lonza (PBS), and cultured in complete medium containing 2.5 μg/ml puromycin from InvivoGen.

### Measurement of cell viability

In 96-well plates, 50 000 cells were incubated at 37°C for 24 hours with increasing concentration of his-TRAIL (from 0 to 10 000 ng/ml) or for 48 hours with increasing concentration of CDDP (from 1 to 1000 μM). Cell viability was determined by methylene blue [22].

### Hoechst analysis

Cells, treated or untreated with His-TRAIL or FasL, were incubated at 37°C for 6 hours. Alternatively, cells pre-treated or not for 30 minutes with 100 μM H89, 1 or 100 μM forskolin or for 20 minutes with 4 mM 8-bromo-cyclic AMP (8B), followed by TRAIL (100 or 500 ng/ml for 6 hours) or Fas ligand (100 ng/ml for 6 hours), were incubated at 37°C for 6 hours. Apoptosis was assessed by Hoechst staining by determining the percentage of condensed and fragmented nuclei from at least 300 cells per conditions. Experiments were repeated at least three times.

### APO 2.7 staining

Cells, treated or untreated with His-TRAIL or FasL, pre-treated or not with 10 μM H89, were permeabilized (PBS, FCS 2,5% and digitonin 100 μg/ml) for 10 min at 4°C and stained with a PE-conjugated 2.7A6A3 antibody (Beckman Coulter) which recognizes the APO2.7 mitochondrial membrane protein exposed at an early stage on cells undergoing apoptosis. In all, 10 000 events were analyzed using a FACScalibur flow cytometer (BD Biosciences).

### Immunoprecipitations

For TRAIL DISC analysis, 10^8^ cells were stimulated with 5 μg of Flag-TRAIL cross-linked with 10 μg of anti-Flag M2 in 1ml of medium for the indicated times at 37°C. Cells were then washed with cold phosphate saline buffer (PBS) and lysed in 1ml of lysis buffer containing 1% of NP40, 20 mM Tris-HCl pH 7.5, 150 mM NaCl and 10% glycerol and proteinase inhibitor cocktail. Lysates were pre-cleared with Sepharose 6B (Sigma-Aldrich), and immunoprecipitated overnight at 4°C with protein G-Sepharose beads (Amersham Biosciences, Les Ullis, France). For TRAIL receptor or GAPDH immunoprecipitations, cells were stimulated as described above with 5 μg/ml of His-TRAIL. In both cases, cells were lysed in NP40-containing lysis buffer. Cell extracts were precleared and immunoprecipitated using 5 μg of corresponding antibodies. Beads were then washed four times with lysis buffer, and immunoprecipitates were eluted in loading buffer (Tris-HCl 63 mM, SDS 2%, phenol red 0.03%, glycerol 10% and DTT 100 mM of pH 6.8), boiled for 5 min and processed for immunoblotting.

### Western blotting

Immunoprecipitates or cell lysates were resolved by sodium dodecyl sulfate-polyacrylamide gel electrophoresis (SDS-PAGE) and transferred to nitrocellulose membranes. Nonspecific binding sites were blocked by incubation in PBS containing 0.05% Tween 20 and 5% milk powder. Membranes were then incubated with a specific primary antibody followed by horseradish peroxidase-conjugated secondary antibody, and were developed by the enhanced chemiluminescence method according to the manufacturer’s protocol (Pierce, Rockford, IL, USA).

### Analysis of Bax activation by flow cytometry

Cells, treated or untreated with His-TRAIL, were fixed with 4% PFA, permeabilized (PBS, BSA 1% and saponin 0.1%) for 10 min at room temperature and stained with an anti-Bax antibody which recognizes the active N-terminal form of Bax (clone 6A7, BD Biosciences). 10 000 events were analyzed using a FACScalibur flow cytometer (BD Biosciences).

### Statistical Analysis

For in vitro studies, differences were determined either with two-way repeated-measures analysis of variance (ANOVA) with Bonferroni's multiple comparison test, or by student's t test, using Prism 5.0a software (GraphPad Software, San Diego, CA, USA). A significance level of *P<0.05, **P<0.01 or ***P<0.001 was assumed for all tests.

## Results

### Ezrin is a negative regulator of Fas and TRAIL-induced cell death

**Fig 1:**
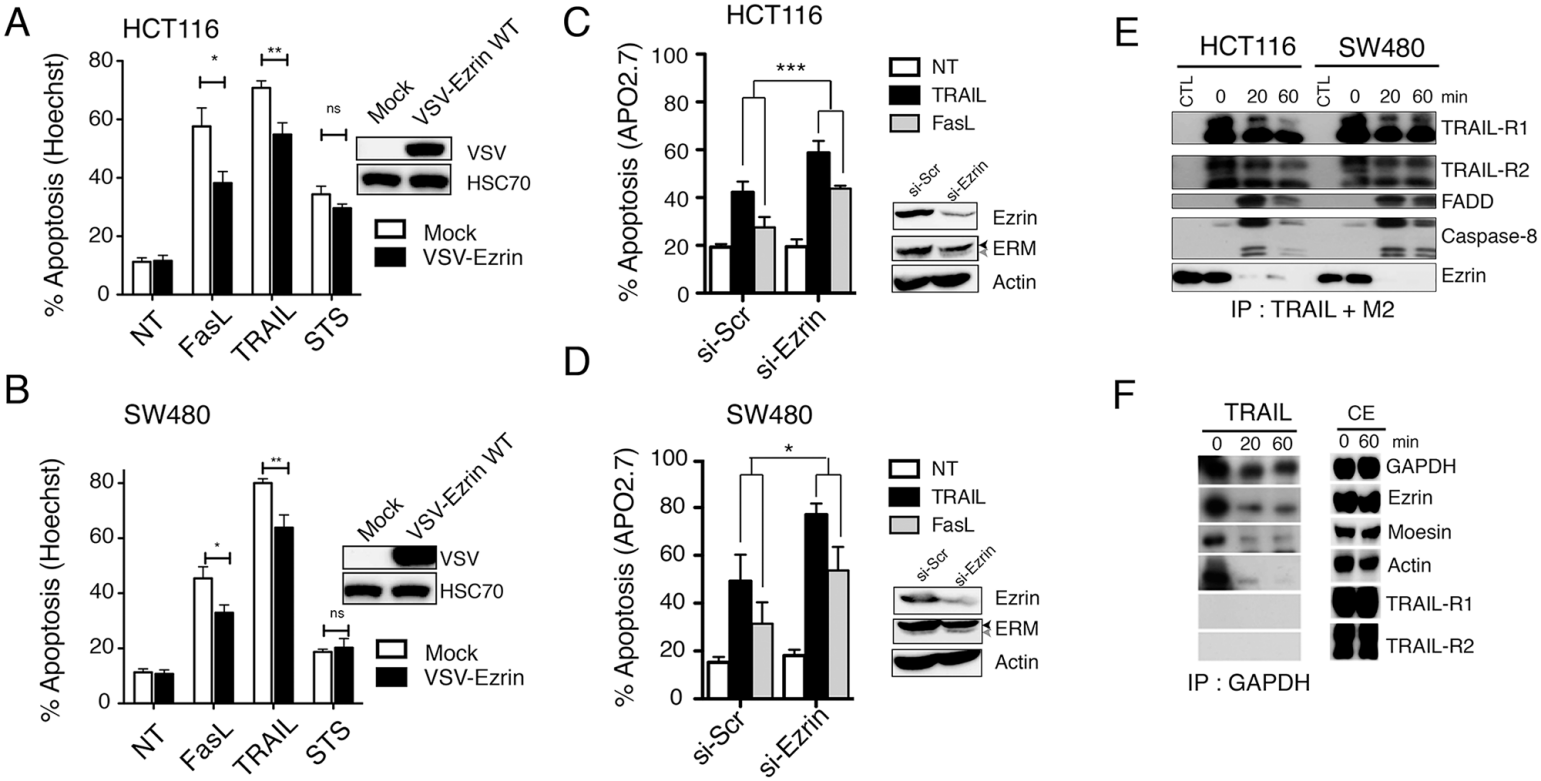
Ezrin inhibits TRAIL-induced apoptosis downstream of the DISC. (A) HCT116 or (B) SW480 cells, expressing or not VSV-ezrin were treated for 6 hours with Fas ligand (100 ng/ml) or His-TRAIL (500 ng/ml) or 16 hours with 1μM staurosporin (STS). Apoptosis was quantified by Hoechst staining. Data represent the mean ± SD of at least three different experiments. (*P<0.05; **P<0.01 respective to control cells). Ezrin ectopic expression levels were analyzed by immunoblotting using an anti-VSV antibody in control or ezrin WT-expressing HCT116 and SW480 cells. HSC70 was used as a loading control. (C) HCT116 or (D) SW480 cells, were transfected ezrin or scramble (Scr) siRNAs. 72 h after transfection cells were stimulated for 6 hours with Fas ligand (100 ng/ml) or His-TRAIL (500 ng/ml) and apoptosis was quantified after staining with APO2.7 antibody by flow cytometry. Data represent the mean ± SD of at least three different experiments. (*P<0.05; **P<0.01 respective to control cells). Ezrin expression levels were analyzed by immunoblotting. Actin was used as a loading control. (E) Analysis of TRAIL DISC formation. HCT116 and SW480 cells were stimulated or not with 5 μg/ml Flag-TRAIL cross-linked with 10 μg/ml anti-Flag (M2) antibody. Cells were lysed, and the DISC was immunoprecipitated and analyzed by western blot. One of three independent experiments is shown. (F) HCT116 cells were stimulated or not with 5 μg/ml His-TRAIL for 20 and 60 minutes. After cell lysis, GAPDH antibody was added to the cell lysates and immunoprecipitates were analyzed by western blot.

Ezrin and moesin were previously demonstrated to be positive regulators of Fas-induced cell death through their ability to interact with Fas receptor in lymphoid T cells [9, 13]. Accordingly, ezrin and moesin silencing in Jurkat cells significantly inhibited FasL-induced apoptosis, and similarly inhibited apoptosis induced by TRAIL (S1 Fig). The role of ezrin in regulating Fas- and TRAIL-induced apoptosis was then evaluated in colon carcinoma cells. Ezrin was either stably overexpressed (Fig 1A and 1B) or silenced using siRNA (Fig 1C and 1D) in HCT116 and SW480 cells and apoptosis-induced by TRAIL or Fas ligand was assessed by Hoechst staining. Ectopic expression of Ezrin significantly attenuated Fas ligand- and TRAIL-induced cell death in the two cell lines (Fig 1A and 1B). Sensitivity to apoptosis induced by staurosporine, a PKC-inhibitor known to induce mitochondrial activation, was however not altered in these cells (Fig 1A and 1B). Consistent with these results, ezrin silencing increased both FasL- and TRAIL-induced apoptosis (Fig 1C and 1D).

### Ezrin is not recruited in the TRAIL DISC

Since ezrin has been demonstrated to be a component of the Fas DISC in lymphoid cells [13, 14], we next checked whether ezrin was recruited in the TRAIL DISC in colon cancer cells. HCT116 or SW480 cells were left unstimulated (CTL and 0) or stimulated with 1 μg/ml rhFlag-TRAIL cross-linked with 2 μg/ml M2 for 15, 20, 30 or 60 minutes. After stimulation, cells were lyzed and the DISC was immunoprecipitated, using sepharose-protein G beads. Control immunoprecipitation was also performed using an irrelevant antibody (CTL) in non-stimulated cells. As shown Fig 1E, TRAIL stimulation induced DISC formation as evidenced by the recruitment of the adaptor protein FADD as well as the initiator caspases-8 and -10 to the TRAIL receptors. Recruitment of ezrin was, however, not consistently observed within the TRAIL DISC, even after selective immunoprecipitation of either TRAIL-R1 (S2 Fig) or TRAIL-R2 (S3 Fig). To check the possibility that ezrin pull-down might be unspecific, a protein not related to the TRAIL or Fas pathway was immunoprecipitated in cells stimulated with another version of TRAIL, the rh-His-TRAIL, a ligand able to induce apoptosis in the absence of M2 cross-linking. Immunoprecipitation of the GAPDH pulled-down similar amounts of ezrin, moesin and actin from non-stimulated cells but less so from lysates obtained from TRAIL stimulated cells (Fig 1F). Altogether these results argue against a physiological function for ezrin at the DISC level in colon cancer cells.

### Ezrin phosphorylation modulates TRAIL-induced cell death

**Fig 2:**
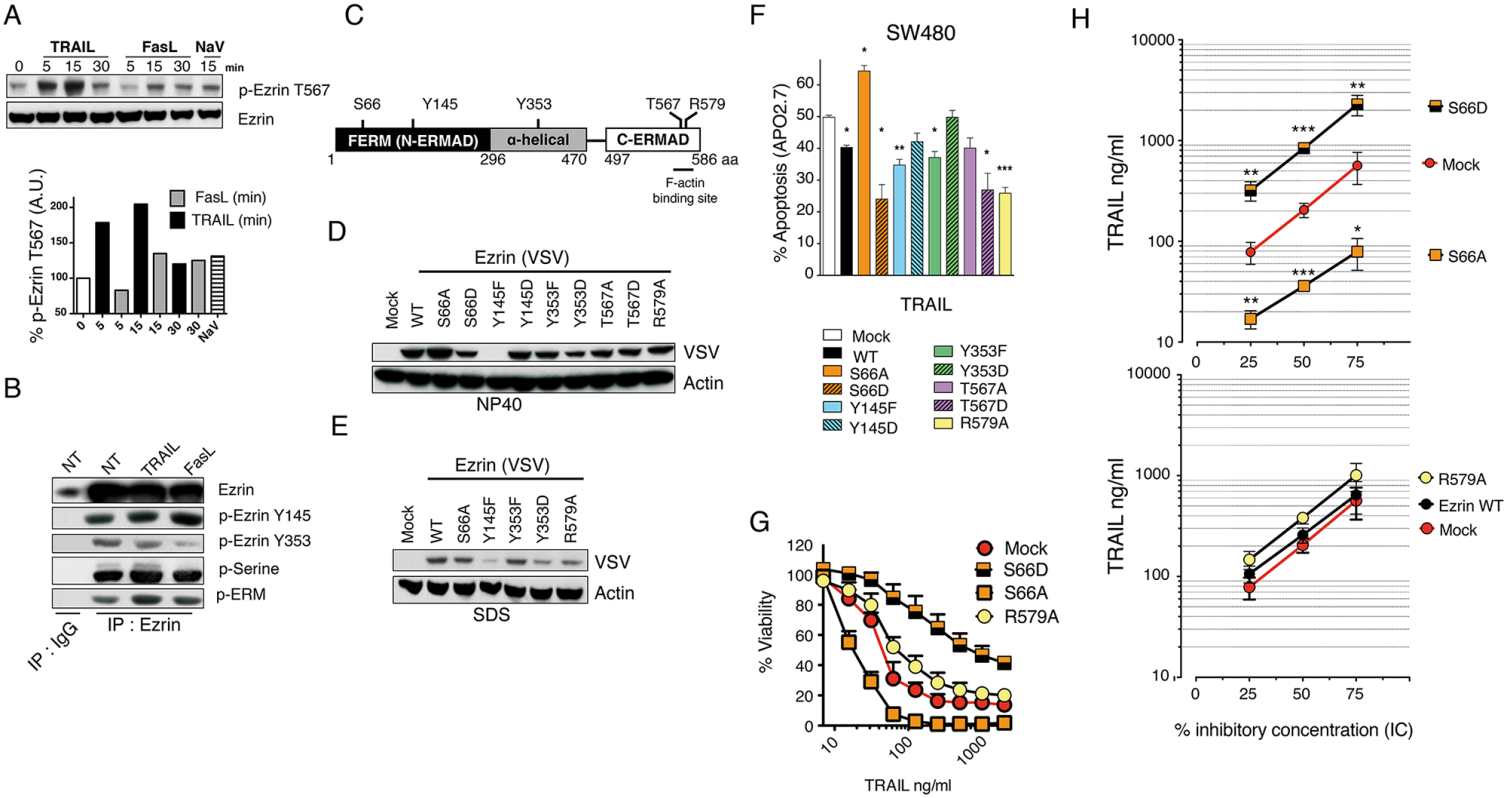
Ezrin phosphorylation on serine 66 differentially affects its ability to inhibit TRAIL-induced apoptosis. (A) Immunoblot analysis of phospho-ezrin (Thr567) expression levels in SW480 cells after stimulation with His-TRAIL, Fas ligand or orthovanadate (NaV). Percentage of relative phospho-ezrin (Thr567) intensities were determined as follows: intensity of specific band in stimulated cells divided by the normalized intensity of unstimulated cells, normalized to HSC70. (B) SW480 cells were stimulated with 500 ng/ml His-TRAIL or 100 ng/ml Fas ligand for 15 minutes or left untreated. After cell lysis in NP40-containing buffer, ezrin was immunoprecipitated with an anti-ezrin antibody (clone 3C12). The level of ezrin phosphorylation was determined by western blot using anti-phospho-ezrin targeting tyrosines 353 and 145, anti-phospho-ERM recognizing phosphorylated-ezrin on threonine 567,-moesin on threonine 558 and-radixin on threonine 564 and an anti-pan phosphoserine. (C) Schematic representation of ezrin domains and phosphorylation sites within the protein. (D) SW480 cells were infected with an empty pMSCV retroviral vector (Mock) or with a pMSCV vector encoding ezrin WT, ezrin S66A, ezrin S66D, ezrin Y145F, ezrin Y145D, ezrin Y353F, ezrin Y353D, ezrin T567A, ezrin T567D and ezrin R579A. Expression levels of ezrin constructs were determined by immunoblot from NP40 cell extracts using an anti-VSV antibody. Actin was used here as a loading control. Data shown is representative of three independent experiments. (E) Selected cell extracts obtained after lysis in SDS were analyzed by immunoblot as above. (F) Effect of ezrin WT and ezrin phosphomutants ectopic expression on TRAIL-induced cell death in SW480 cells. Cells were stimulated with TRAIL 500 ng/ml for 6 hours. Apoptosis was measured by APO2.7 staining by flow cytometry. (G) Cell viability in the SW480 cells expressing ezrin S66A, ezrin S66D or ezrin R579A as compared to Mock infected cells was evaluated by methylene blue assay 24h after treatment using increasing concentrations of His-TRAIL. (H) Percent inhibitory TRAIL concentration curves, in ng/ml, from SW480 cells expressing ectopically the indicated ezrin mutants were obtained by methylen blue staining 16h after increasing His-TRAIL concentrations. Corresponding IC25, IC50 and IC90, inducing 25, 50 and 90% cell death, were obtained using CompuSyn. Data represent mean ± SD of at least 3 independent experiments. *P<0.05; **P<0.01; ***P<0.001 respective to Mock control cells.

Regulation of ezrin phosphorylation was proposed to account for its ability to interfere with Fas signalling [13, 23]. Like FasL, TRAIL induced an increase in ezrin phosphorylation on threonine 567 in SW480 cells (Fig 2A). Moreover, as evidenced after ezrin immunoprecipitation, an increase in ezrin tyrosine 145 and serine residues phosphorylation was also found after TRAIL stimulation (Fig 2B). On the other hand, however, phosphorylation of ezrin tyrosine 353 was found to be slightly reduced in cells stimulated with TRAIL, but to a lower extent as compared to cells stimulated with FasL (Fig 2B).

In order to determine whether ezrin phosphorylation [19, 20, 24] affects TRAIL signalling, we next generated several phosphorylation mutants encoding nonphosphorylatable variants or pseudophosphorylated variants of ezrin, at serine 66, threonine 567 and tyrosines 145 and 353 sites by site directed mutagenesis (Fig 2C). In addition to these phosphorylation mutants, an ezrin mutant defective in F-actin binding, ezrin R579A was generated to determine the role of actin cytoskeleton in ezrin-mediated TRAIL inhibition [25]. Infection of SW480 cells with a retroviral vector encoding these constructs led to variable but appreciable expression levels of ezrin mutants (Fig 2D), with the exception of the nonphosphorylatable variant Y145F, that was mostly expressed in the insoluble fraction (Fig 2E). Interestingly most ezrin mutants impaired TRAIL- (Fig 2F) and Fas ligand-induced apoptosis (S4 Fig). Remarkably, the nonphosphorylatable variant S66A significantly and selectively enhanced apoptosis induced by TRAIL whereas the pseudophosphorylated variant S66D demonstrated superior protective effect as compared to ezrin WT and to mock cells (Fig 2F and 2G). Alteration of S66 phosphorylation, however, failed to regulate ezrin-mediated FasL-induced apoptosis inhibition (S4 Fig). Like WT ezrin, the actin-binding deficient mutant R579A inhibited apoptosis induced by FasL and TRAIL (Fig 2F and S4 Fig), suggesting that inhibition of apoptosis induced by death receptor ezrin in colon cancer cells can occur independently of the cytoskeleton-binding properties of ezrin. Alteration of ezrin phosphorylation on serine 66 appears to be by far the most important event controlling ezrin's ability to inhibit TRAIL signalling.

As compared to parental cells in which around 200 ng/ml TRAIL are required to induce cell death in 50% of the cells, ezrin WT or R579A mutant expressing cells displayed an IC50 of 260 and 380 ng/ml, while the IC50 in S66A expressing cells was less than 40 ng/ml and that of S66D reached 850 ng/ml (Fig 2G). In other words, regulation of ezrin phosphorylation on S66 modulates TRAIL-induced cell death sensitivity by an order of magnitude ranging from 0.2 to more than 4 fold as compared to parental cells (Fig 2H and S2 Table, for IC% values).

### Ezrin inhibits TRAIL-induced cell death downstream of the TRAIL DISC

**Fig 3:**
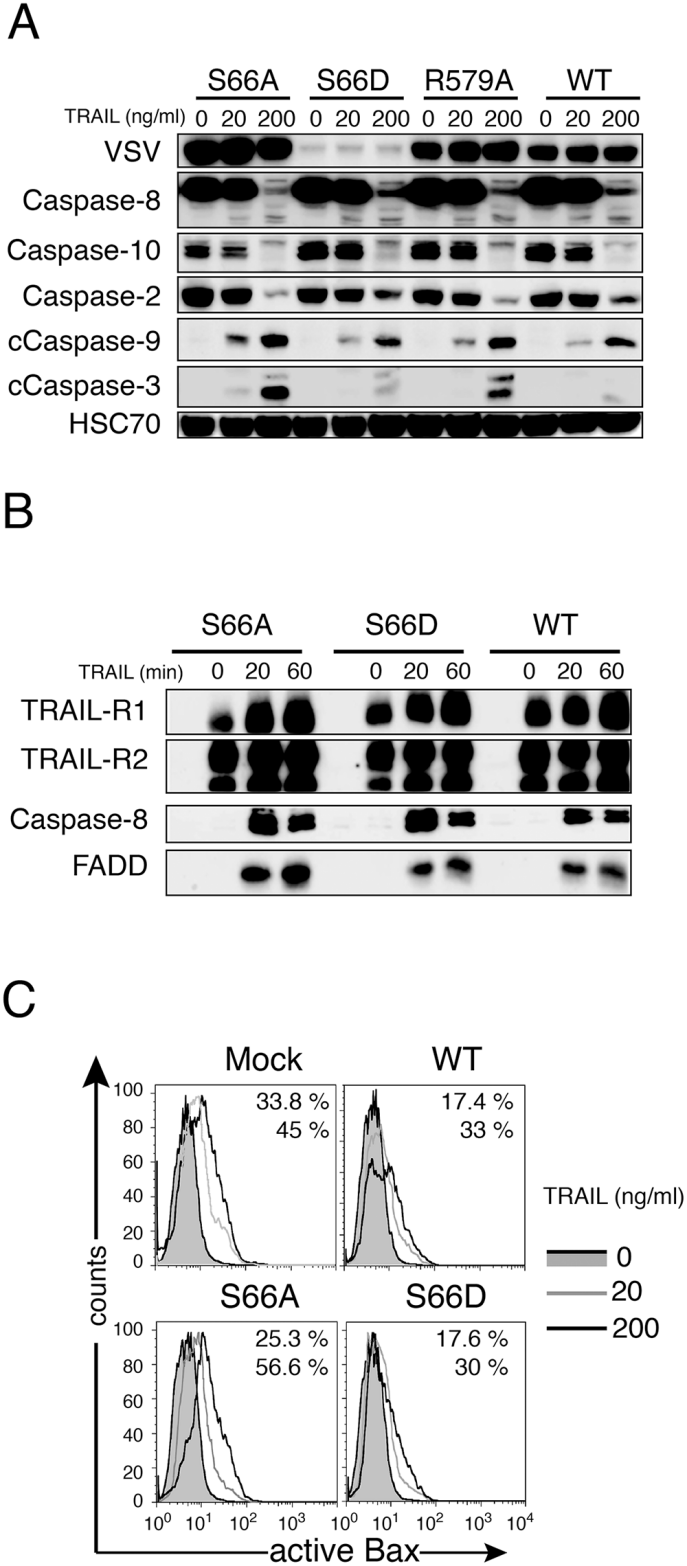
Ezrin inhibits TRAIL-induced cell death at the mitochondria level. (A) Immunoblot analysis of caspase activation, in SW480 cells expressing either ezrin S66A, S66D, R579A or WT, 16 or 6 hours after His-TRAIL (20 or 200 ng/ml) stimulation, respectively. (B) Analysis of TRAIL DISC formation in SW480 cells expressing either S66A, S66D, or WT ezrin. Cells were stimulated with 5 μg/ml Flag-TRAIL cross-linked with 10 μg/ml anti-Flag (M2) antibody. After cell lysis, the DISC was immunoprecipitated and analyzed by western blot. (C) Analysis of Bax activation. Ezrin S66A, S66D, WT and Mock-infected SW480 cells were left untreated or stimulated with His-TRAIL (20 or 200 ng/ml) for 16 h, then permeabilized and stained with an antibody recognizing active Bax before analysis by flow cytometry. The effect of two different concentrations of TRAIL (20 or 200 ng/ml, gray and black lines, respectively) were compared to unstimulated cells (gray filled curve). The percentage of cells containing active Bax after TRAIL stimulation is shown (upper and lower value, respectively).

Analysis of caspase-8 and caspase-10 activation in cell lysates obtained from SW480 cells expressing either ezrin WT, R579A, S66A or S66D, stimulated with increasing concentrations of TRAIL, revealed that TRAIL DISC associated initiator caspases were activated in a similar fashion, irrespective of the ezrin mutant (Fig 3A). On the other hand, activation of caspase-9 and caspase-3 were slightly enhanced in ezrin S66A expressing SW480 cells and consistently reduced in SW480 cells expressing ezrin WT, ezrin S66D and ezrin R579A, as jugged from the corresponding cleaved products (Fig 3A). Moreover, TRAIL DISC formation was neither altered by ectopic expression of ezrin S66A, nor S66D mutants (Fig 3B), and none of the ezrin mutant that we have tested so far were able to change TRAIL-R1 or TRAIL-R2 expression levels (S5 Fig), indicating that ezrin's regulatory activity occurs downstream TRAIL DISC, most likely at the level of the mitochondria. Supporting this conclusion was the finding that Bax activation in response to 200 ng/ml TRAIL stimulation dropped from 45% to 30% in cells expressing ezrin WT or ezrin S66D as compared to mock-transfected cells (Fig 3C), whereas, activation of Bax in S66A ezrin expressing cells reached nearly 57% of the cells after stimulation (Fig 3C).

### Ezrin-mediated TRAIL regulation is associated with WWOX

**Fig 4:**
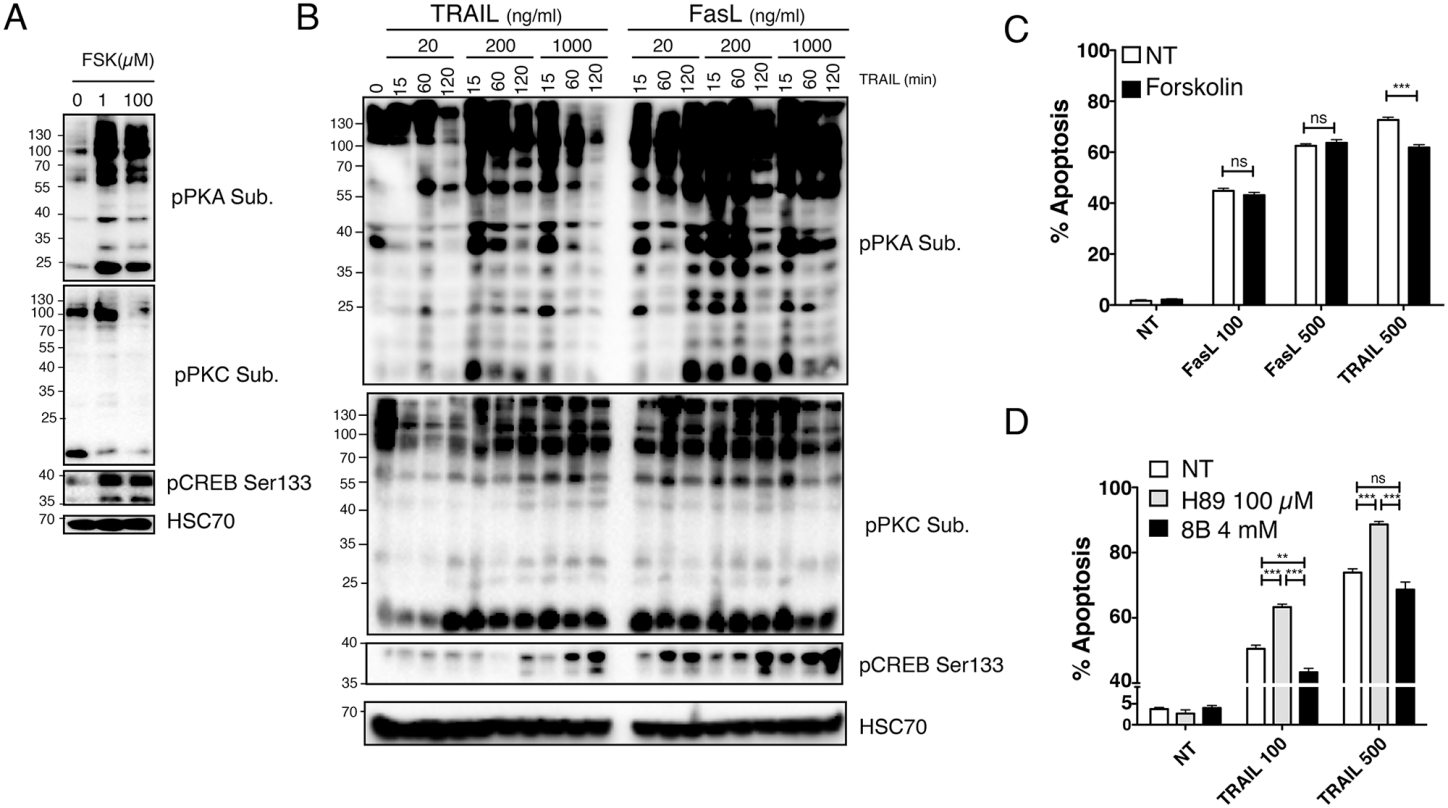
Induction of PKA activity by TRAIL restrains its pro-apoptotic potential. (A) Parental SW480 cells were stimulated for 30 minutes with forskolin and cell extracts were analyzed by immunoblot using selective PKA or PKC substrate antibodies as well as an anti-phospho-S133 CREB antibody. (B) Parental SW480 cells were analyzed as above after stimulation with TRAIL or FasL as indicated for 15 to 120 minutes. (C) Parental SW480 cells were pre-treated or not for 30 minutes with indicated concentrations of forskolin, followed by 6 hours stimulation with 100 or 500 ng/ml FasL or TRAIL. Apoptosis was quantified by Hoechst staining. (D) Parental SW480 cells were pre-treated or not for 30 minutes with 100 μM H89 or 20 minutes with 4 mM 8B, followed by 6 hours stimulation with 100 or 500 ng/ml TRAIL. (C and D) Data represent the mean ± SD of at least three different experiments. (**P<0.01; ***P<0.001 respective to control or H89 stimulated cells; ns stands for not statistically relevant).

**Fig 5:**
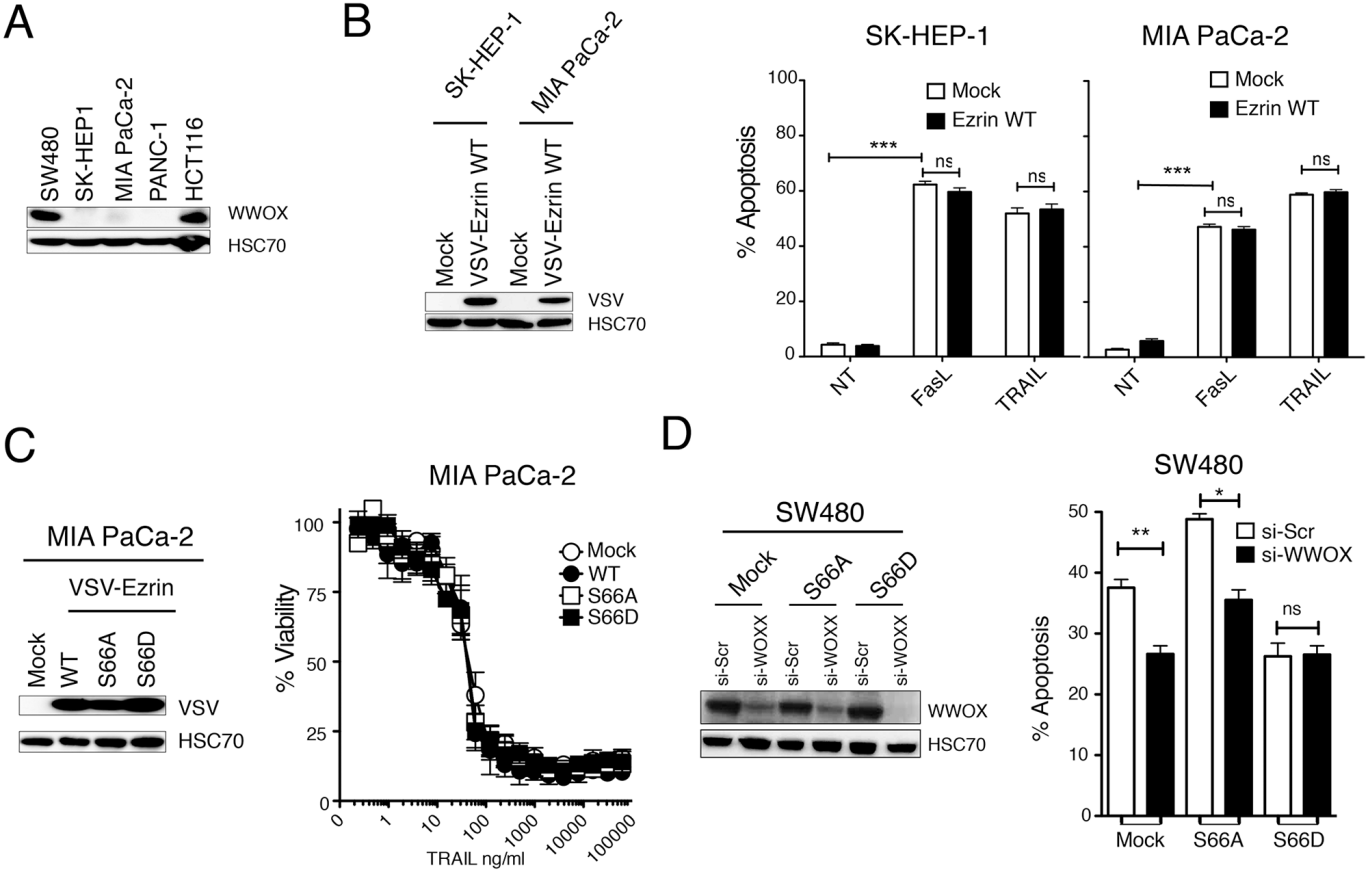
Ezrin-mediated TRAIL inhibition involves WWOX. (A) WWOX expression levels in indicated cell lines were analyzed by immunoblot. HSC70 was used here as a loading control. (B) VSV-ezrin WT was expressed ectopically in WWOX-deficient cells SK-HEP-1 and MIA PaCa-2. Expression levels were analysed by immunoblot and apoptosis in the corresponding cell lines after FasL (100 ng/ml) or TRAIL (500 ng/ml) stimulation was analysed by Hoechst staining. (C) S66A and S66D ezrin mutants were expressed in MIA PaCa-2. Expression levels were analysed by immunoblot as above and cell sensitivity to TRAIL-induced cell death was quantified by methylene blue. (D) SW480 cells expressing either ezrin wt or ezrin S66A or S66D were transfected with scramble (Scr) or WWOX (WOXX) si-RNAs for 4 hours and sensitivity to apoptosis induced by TRAIL was quantified by Hoechst staining. Data represent the mean ± SD of at least three different experiments. (**P<0.01; *P<0.05 respective to Scr siRNA tranfected cells; ns stands for not statistically relevant).

Protein kinase A has been shown to mediate the phosphorylation of ezrin on serine 66 [20]. Like forskolin, an activator of PKA, TRAIL induced activation of PKA, but also PKC, as evidenced using antibodies recognizing PKA and PKC substrates (Fig 4A and 4B). Consistent with PKA activation, phosphorylation of CREB on serine 133 increased in dose and time dependent manner in these cells (Fig 4B), and pharmacological modulation of PKA in SW480 cells phenocopied the regulation of TRAIL-induced apoptosis by the S66 ezrin mutants (Fig 4C and 4D). Likewise, inhibition of PKA, by use of the inhibitor H89, mimicked the effects of ezrin S66A expression, albeit to a lower extent, and increased TRAIL-induced apoptosis, while activation of PKA using forskolin or 8-Bromoadenosine 3′,5′-cyclic monophosphate (8B), reduced TRAIL-induced apoptosis in SW480 cells (Fig 4C and 4D). It should be noted here that consistent with results obtained with ezrin serine 66 phosphorylation mutants and its strongest ability to trigger PKA activation (Fig 4B), Fas ligand-induced cell death, contrary to TRAIL, was not significantly altered by pharmacological PKA regulators (Fig 4C and S6 Fig). PKA-induced phosphorylation of ezrin on S66 has been shown to regulate its interaction with WWOX (**WW domain-containing oxidoreductase)** [26], a tumor suppressor protein [27] that regulates apoptosis induced at the mitochondrial level [28]. Interestingly, whereas both colon carcinoma cell lines SW480 and HCT116 express WWOX, two pancreatic cell lines, MIA PaCa-2 and PANC-1 as well as the SK-HEP-1 liver cancer cell line (Fig 5A) do not, and ectopic expression of ezrin wt in MIA PaCa-2 or SK-HEP-1 failed to regulate apoptosis induced by FasL or TRAIL (Fig 5B). Moreover, neither S66A nor S66D ezrin mutants, which in SW480 cells exhibit the most striking regulatory phenotype, modulated apoptosis induced by TRAIL (Fig 5C). On the other hand, WWOX silencing inhibited apoptosis induced by TRAIL both in Mock and S66A SW480 expressing cells, but not in cells expressing S66D (Fig 5D). These results suggest that the impact of ezrin in death receptor-induced apoptosis is most likely indirect and that regulation of ezrin phosphorylation on serine 66 by PKA is likely to target WWOX pro-apoptotic potential, explaining at least in part ezrin's ability to control apoptosis induced by death receptors.

## Discussion

While the contribution of ezrin in Fas signalling has been extensively studied, little is known regarding TRAIL, with the exception of a recent study in which ezrin was proposed to impair both Fas ligand- and TRAIL-induced cell death in the tumor T-cell lymphoma cell line H9 [14]. In that study, ezrin was suggested to inhibit death receptor-mediated cell death in type I cells, which are independent of the mitochondrial pathway, but not in type II cells [14] that rely on the mitochondria [29]. In our hands, ezrin's negative regulatory function was not strictly restricted to type I cells, since Fas ligand- and TRAIL-induced cell death were inhibited by ezrin overexpression both in SW480 and in HCT116 cells, considered as type I and type II, respectively.

Ezrin-mediated TRAIL-induced cell death regulation was neither related to changes in TRAIL DISC component recruitment nor to differential activation of initiator caspases within the DISC, including caspase-8. Contrary to Fas itself [30, 31], TRAIL signalling regulation by ezrin was also neither associated with variations in receptor steady state membrane expression levels nor with changes in receptor internalization after TRAIL stimulation. Rather, ezrin appeared to target the apoptotic machinery downstream of the TRAIL DISC as demonstrated by its ability to interfere with Bax activation or caspase-9 and caspase-3 cleavage, but not caspase-8 or caspase-10.

The pleiotropic regulatory function of ezrin was uncovered by the analysis of ezrin phosphorylation mutants targeting several major phosphorylation residues including serine 66, threonine 567, tyrosine 145 or tyrosine 353. Ectopic expression of these ezrin mutants in SW480 cells allowed us to demonstrate that ezrin serine 66 phosphorylation or dephosphorylation contributes to TRAIL-induced cell death regulation but not to apoptosis triggered by Fas ligand or staurosporine. Remarkably, while S66D phosphomimetic ezrin mutant induced strong protection against TRAIL-induced cell death, the corresponding nonphosphorylatable mutant S66A increased cell sensitivity to TRAIL. Ezrin phosphorylation on threonine 567, although reported as a prerequisite for Fas aggregation and caspase-8 activation [13], did not enhance Fas ligand- nor TRAIL-induced cell death in colon cancer cells. Importantly, execution of the apoptotic machinery was associated with changes in caspase-3 and Bax activation. These results indicate that ezrin regulates TRAIL- or Fas ligand-induced cell death irrespective of its ability to interact with the corresponding death receptors in colon cancer cells. In line with this hypothesis, expression of ezrin R579A, an ezrin mutant defective in F-actin binding, inhibited both TRAIL- and Fas ligand-induced apoptosis as efficiently as ezrin WT or ezrin mutants targeting threonine 567 and tyrosine 145.

How regulation of ezrin phosphorylation on serine 66 differentially affects TRAIL-induced cell death is unclear. ERM proteins, including ezrin, predominantly exist in a “dormant” cytosolic form, folded by intramolecular association of their N and C termini, but are activated or unfolded through phosphatidylinositol 4,5-bisphosphate (PIP_2_) binding and phosphorylation [16]. It is therefore tempting to speculate that ezrin phosphorylation on serine 66 gives rise to similar ezrin conformational changes inducing specific inhibition of TRAIL signalling, while dephosphorylation of serine 66 would lead to another conformation enhancing execution of the apoptotic program. Alternatively, phosphorylation or dephosphorylation of these residues is likely to disrupt or favour interactions with unknown or known ezrin protein partners, some of which may play a role in regulating apoptosis induced by death receptors. In line with this hypothesis we show here that TRAIL was able to induce activation of PKA and that pharmacological agents inhibiting or activating PKA activity phenocopied point mutations of ezrin on serine 66, and regulated either positively or negatively TRAIL-induced cell death in colon cancer cells. The lack of regulatory activity of the different PKA pharmacological regulators used in our study, as well as the S66-non-phosphorylatable mutant, upon Fas ligand stimulation, may be explained by its ability to induce stronger PKA activation, as compared to TRAIL. Yet other signalling events may also contribute to this divergent regulatory function associated with phosphorylation of ezrin on serine 66, including differential activation of phosphatases or kinases leading to differential ezrin phosphorylation. Likewise, phosphorylation of ezrin on T567 or on serines was substantially different in cells stimulated with TRAIL as compared to FasL (Fig 2), suggesting that activation of PKA activity by these ligands is likely to affect ezrin interaction with WWOX. Althought it has been demonstrated that PKA-mediated ezrin phosphorylation at serine 66 is required for ezrin to interact [26] with the tumor suppressor protein WWOX in gastric parietal cells [27], we have not been able to show this interaction in our cell lines, irrespective of the stimuli. Nonetheless we could demonstrate that WWOX silencing blunted sensitization to TRAIL-induced cell death in S66A expressing SW480 cells and that loss of WWOX expression in the liver and pancreatic cancer cell lines prevented TRAIL signalling regulation by ectopic stable expression of ezrin, irrespective of its phosphorylation status on serine 66. WWOX requirement for ezrin-mediated TRAIL signalling regulation, as evidenced here, most likely provides a good explanation to the lack of regulatory function of ezrin in pancreatic or liver cancer cell lines, as opposed to colon cancer cells. Interestingly, WWOX expression levels are mostly found to be down regulated in liver and pancreatic tumours whereas they are significantly increased in colon cancer cells, as compared to normal cell counterpart (S7 Fig).

Our results demonstrate for the first time that TRAIL-induced cell death regulation by ezrin in colon carcinoma cells requires WWOX and occurs downstream of the TRAIL DISC, through regulation of ezrin phosphorylation on serine 66, owing to TRAIL's ability to induce PKA activation. Further work will be needed to define the molecular interplay between ezrin phosphorylation on serine 66, WWOX interaction and the gain or loss of mitochondrial pro-apoptotic potential induced by TRAIL.

## Supporting Information

S1 Fig Small interfering RNA-mediated ezrin downregulation. 48 hours after transfection with control, moesin or ezrin siRNAs, Jurkat cells were stimulated with 200 ng/ml His-TRAIL or 100 ng/ml Fas ligand for 6 hours. (A) Expression levels of ezrin and moesin was analysed by immunoblot and (B) apoptosis was quantified by flow cytometry after staining with APO2.7. Data represents mean ± SD of three different experiments. (***P<0.001; *P<0.05 respective to Scr siRNA tranfected cells).
(TIF)

S2 Fig Ezrin is not pull-down specifically in TRAIL-R1 DISC. HCT116 and SW480 cells were stimulated or not with His-TRAIL (5 μg/ml) and lysed. Cell lysates were immunoprecipitated with an anti-TRAIL-R1 antibody and analyzed by western blot. One of three independent experiments is shown.
(TIF)

S3 Fig Ezrin is not pull-down specifically in TRAIL-R2 DISC. HCT116 and SW480 cells were stimulated or not with His-TRAIL (5 μg/ml) and lysed. Cell lysates were immunoprecipitated with an (A) anti-TRAIL-R2 antibody and analyzed by western blot. One of three independent experiments is shown.
(TIF)

S4 Fig S66A Ezrin phosphorylation mutant fails to enhance FasL- induced cell death. Effect of ezrin WT or ezrin phosphorylation mutants expression on Fas ligand-induced cell death in SW480 cells. Cells were stimulated with Fas ligand 100 ng/ml for 6 hours and apoptosis was measured by flow cytometry after APO2.7 staining. Data represent mean ± SD of at least 3 independent experiments (*P<0.05; **P<0.01; ***P<0.001 respective to Mock control cells).
(TIF)

S5 Fig The PKA inhibitor H89 fails to enhance FasL- induced cell death in SW480 cells. Parental SW480 cells were pre-treated or not for 30 minutes with 20 or 100 μM H89, followed by 6 hours stimulation with 100 or 500 ng/ml FasL or TRAIL. Data represent the mean ± SD of at least three different experiments. (**P<0.01; ***P<0.001 respective to control cells; ns stands for not statistically relevant).
(TIF)

S6 Fig Expression levels of TRAIL-R1 and TRAIL-R2 are not altered by ectopic expression of Ezrin phosphorylation mutants. Expression levels of agonistic TRAIL receptors were quantified by flow cytometry in HCT116 or SW480 cells expressing ezrin WT as compared to Mock-infected cells. (C) Flow cytometry analysis of TRAIL-R1 or TRAIL-R2 expression levels in SW480 cells expressing ezrin phosphomutants-expressing (unfilled histograms) as compared to Mock infected cells (filled histograms).
(TIF)

S7 Fig Meta-analysis of WWOX mRNA expression in Pancreatic, liver or colon cancers compared to normal cells. The Oncomine (Compendia Bioscience, Ann Arbor, MI) database (http://www.oncomine.org/) was used to determine up-regulation or down-regulation of WWOX in pancreatic (10 datasets), liver (7 datasets) and colorectal cancers (27 datasets) versus normal. In pancreatic and Liver cancer cells WWOX was down-regulated in 13 out of 17 datasets as compared to normal cells, but up-regulated in only 3 datasets. In colorectal cancer cells, on the other hand, WWOX was up-regulated in 15 out of 27 datasets and down-regulated in 2 datasets only. WOXX median rank and p-Values are shown on the left for each tumour type.
(TIF)

S1 Table List of primers used to generate ezrin phosphorylation mutants. (XLS)

S2 Table Calculated TRAIL inhibitory concentrations in ezrin phosphomutants-expressing SW480 cells, using CompuSyn. IC25, IC50 and IC75 percent values correspond to the mean of 4 independent experiments.
(XLS)
