# Supplementary figures and images for: Death Receptor-Induced Apoptosis Signalling Regulation by Ezrin Is Cell Type Dependent and Occurs in a DISC-Independent Manner in Colon Cancer Cells
Source: PLoS One. 2015 May 26;10(5):e0126526. doi: 10.1371/journal.pone.0126526 (PMC4444253; doi:10.1371/journal.pone.0126526)

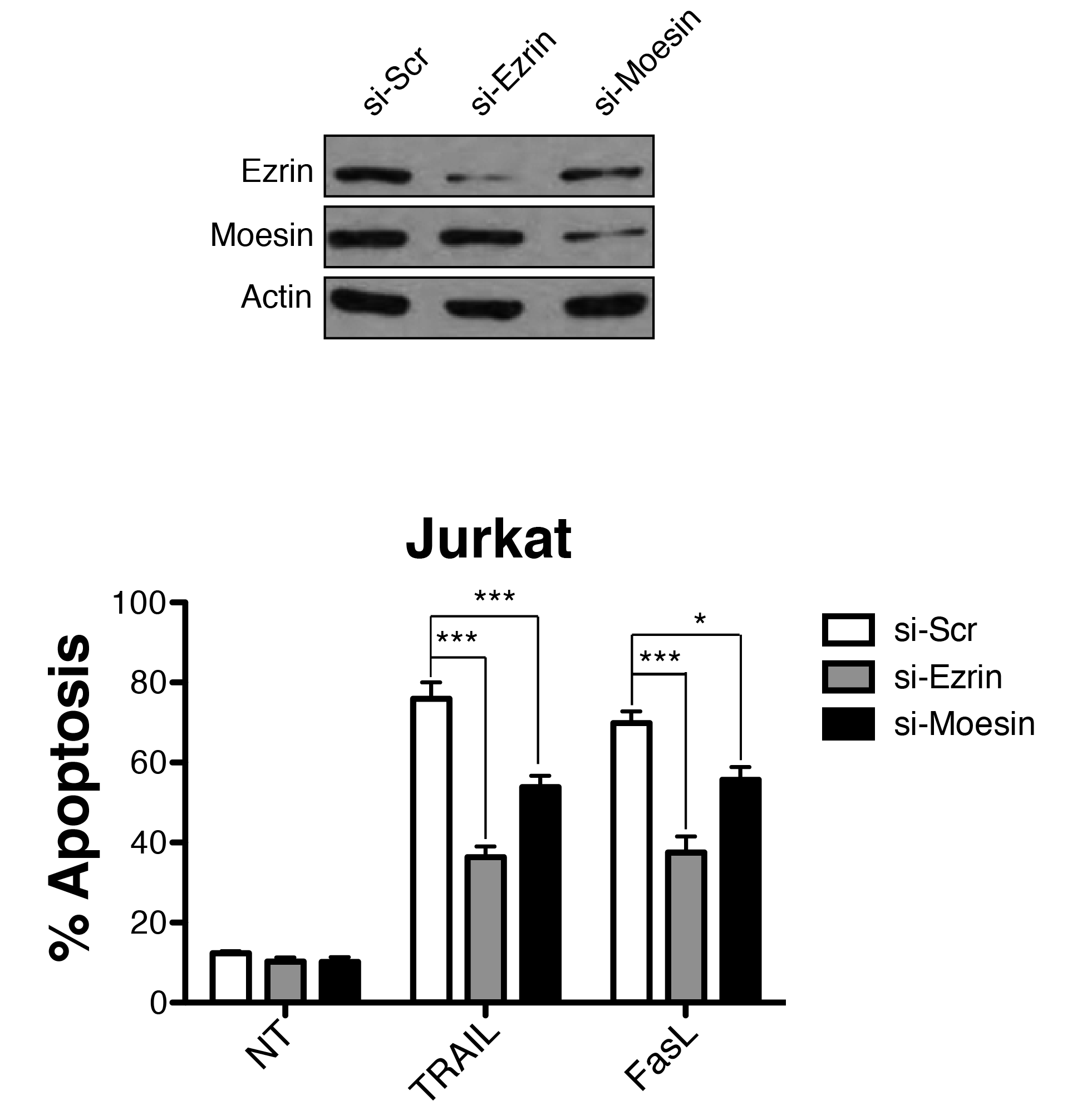

Supplement: S1 Fig — 48 hours after transfection with control, moesin or ezrin siRNAs, Jurkat cells were stimulated with 200 ng/ml His-TRAIL or 100 ng/ml Fas ligand for 6 hours. (A) Expression levels of ezrin and moesin was analysed by immunoblot and (B) apoptosis was quantified by flow cytometry after staining with APO2.7. Data represents mean ± SD of three different experiments. (***P<0.001; *P<0.05 respective to Scr siRNA tranfected cells). (TIF) [file pone.0126526.s001.tif]

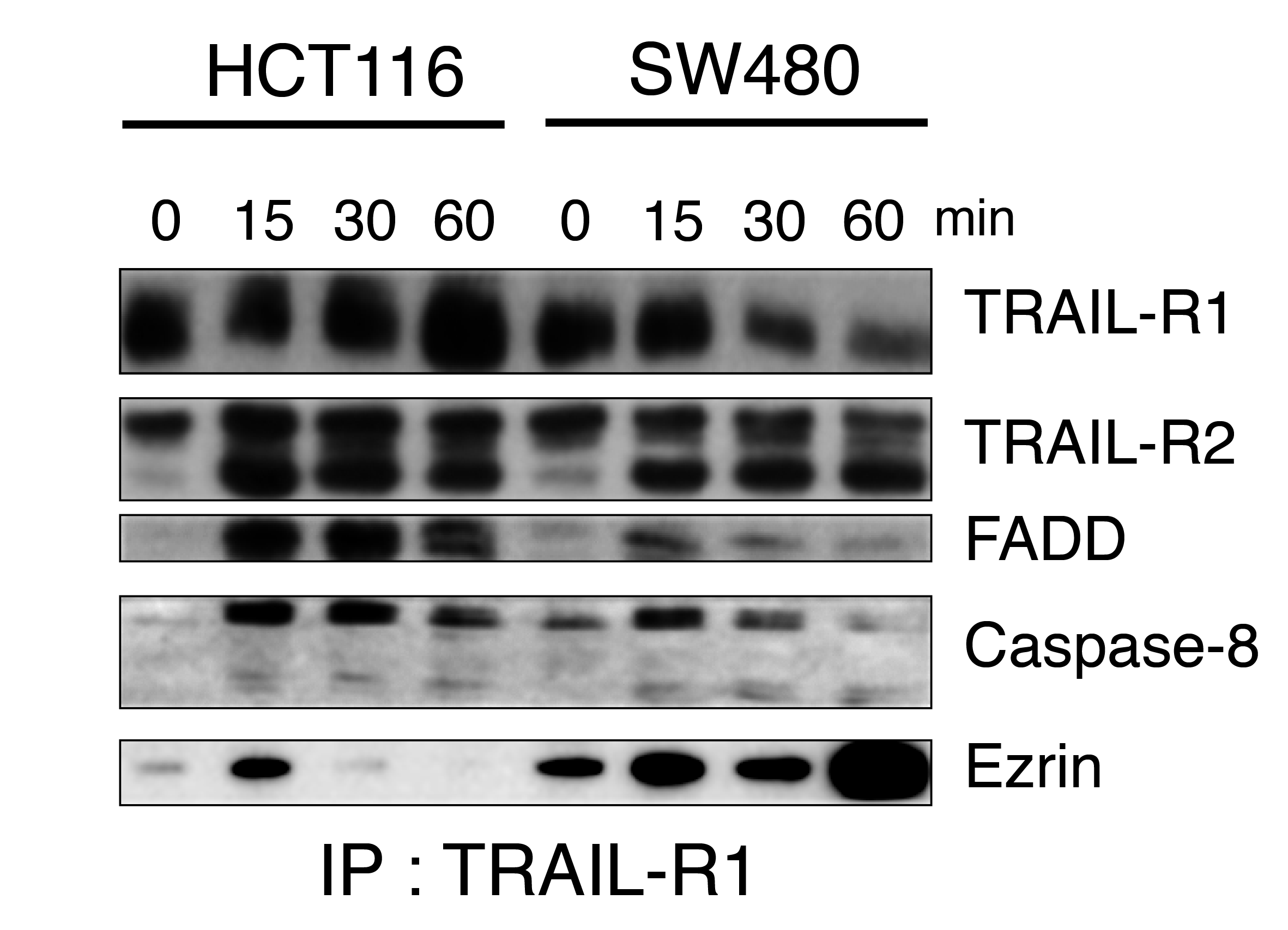

Supplement: S2 Fig — HCT116 and SW480 cells were stimulated or not with His-TRAIL (5 μg/ml) and lysed. Cell lysates were immunoprecipitated with an anti-TRAIL-R1 antibody and analyzed by western blot. One of three independent experiments is shown. (TIF) [file pone.0126526.s002.tif]

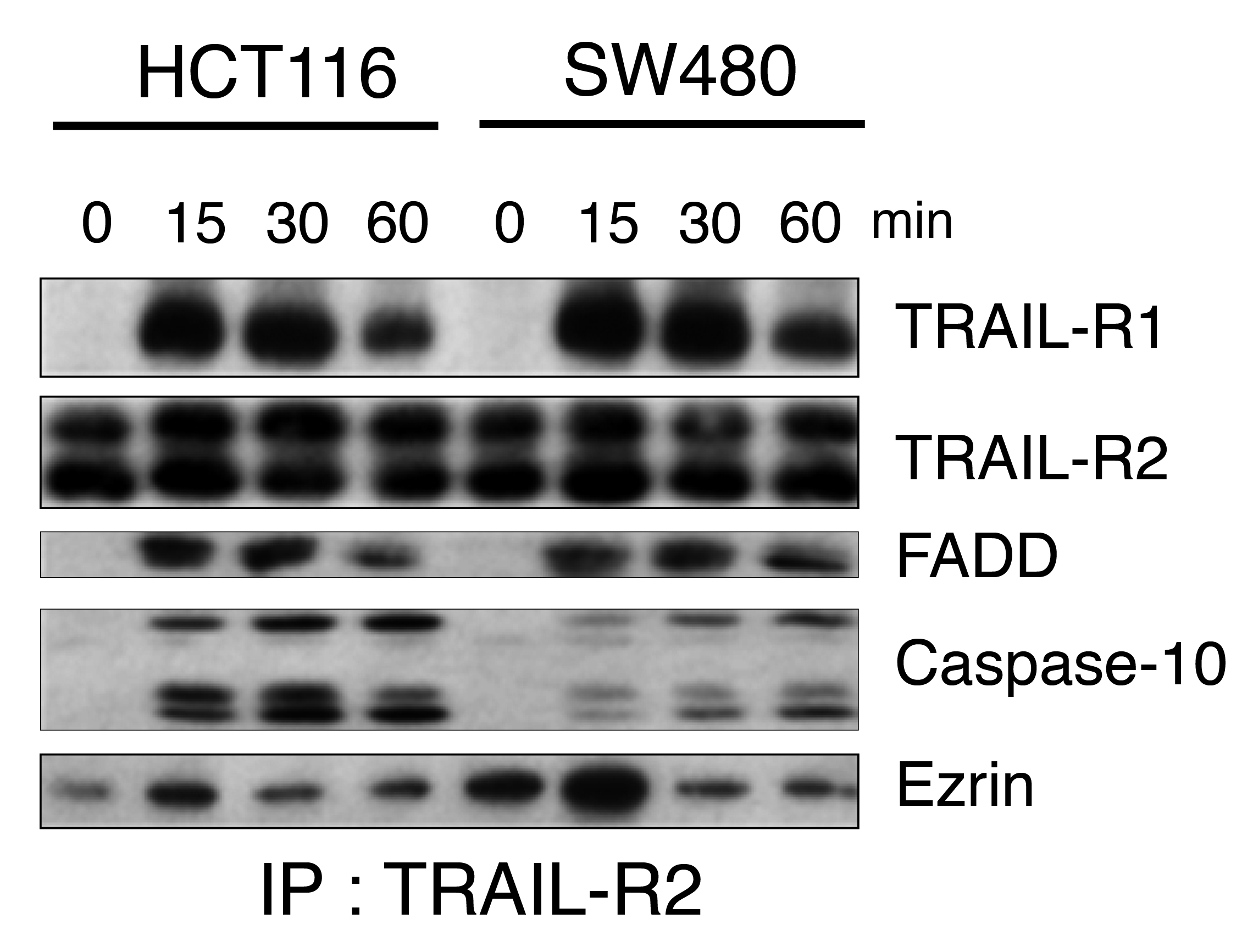

Supplement: S3 Fig — HCT116 and SW480 cells were stimulated or not with His-TRAIL (5 μg/ml) and lysed. Cell lysates were immunoprecipitated with an (A) anti-TRAIL-R2 antibody and analyzed by western blot. One of three independent experiments is shown. (TIF) [file pone.0126526.s003.tif]

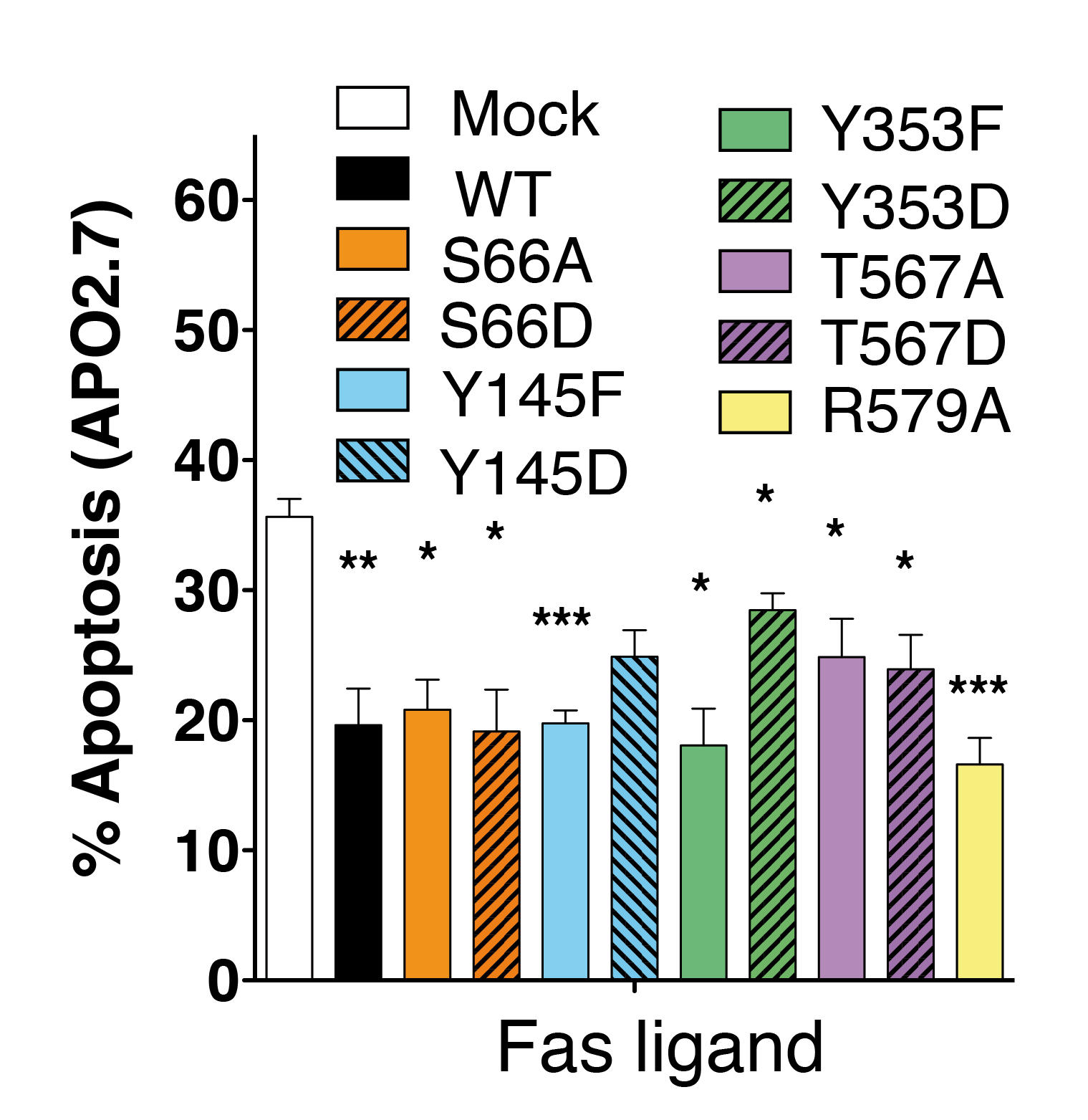

Supplement: S4 Fig — Effect of ezrin WT or ezrin phosphorylation mutants expression on Fas ligand-induced cell death in SW480 cells. Cells were stimulated with Fas ligand 100 ng/ml for 6 hours and apoptosis was measured by flow cytometry after APO2.7 staining. Data represent mean ± SD of at least 3 independent experiments (*P<0.05; **P<0.01; ***P<0.001 respective to Mock control cells). (TIF) [file pone.0126526.s004.tif]

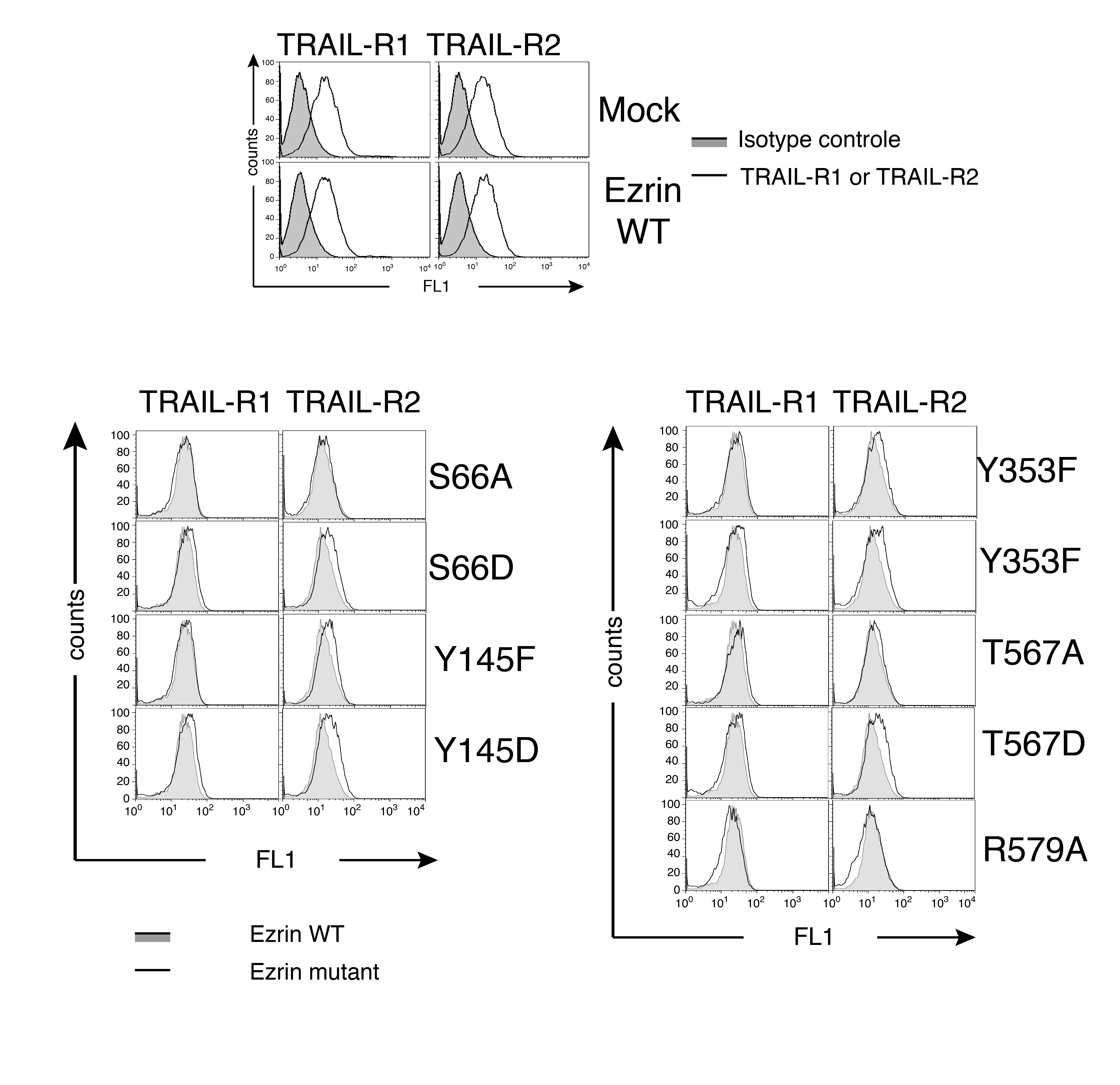

Supplement: S5 Fig — Parental SW480 cells were pre-treated or not for 30 minutes with 20 or 100 μM H89, followed by 6 hours stimulation with 100 or 500 ng/ml FasL or TRAIL. Data represent the mean ± SD of at least three different experiments. (**P<0.01; ***P<0.001 respective to control cells; ns stands for not statistically relevant). (TIF) [file pone.0126526.s005.tif]

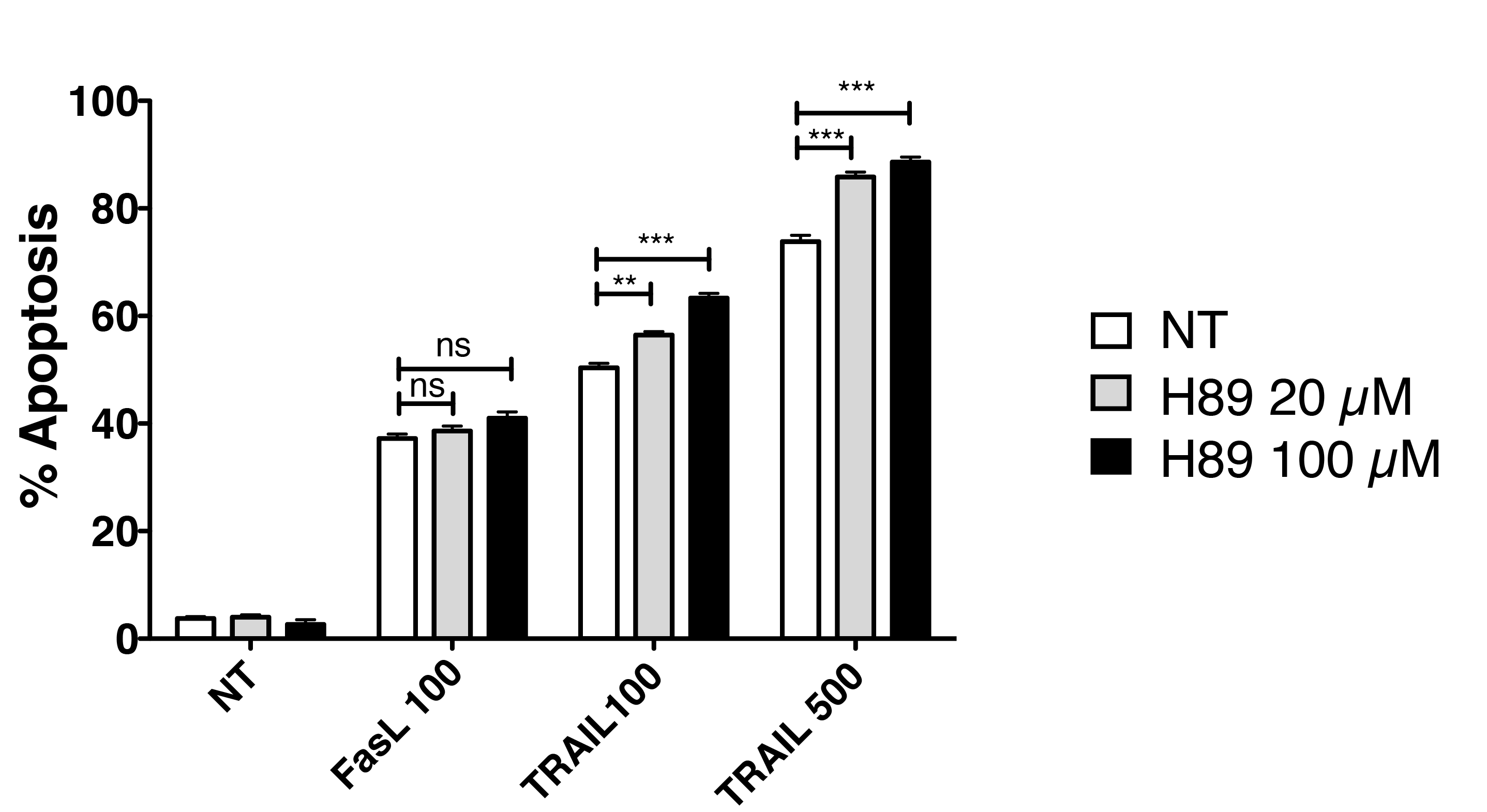

Supplement: S6 Fig — Expression levels of agonistic TRAIL receptors were quantified by flow cytometry in HCT116 or SW480 cells expressing ezrin WT as compared to Mock-infected cells. (C) Flow cytometry analysis of TRAIL-R1 or TRAIL-R2 expression levels in SW480 cells expressing ezrin phosphomutants-expressing (unfilled histograms) as compared to Mock infected cells (filled histograms). (TIF) [file pone.0126526.s006.tif]
